# 

**Published:** 1878-03-20

**Authors:** 


### OFFICE OF THE SOUTHERN MEDICAL RECORD,
**ATLANTA, GA., February, 1878.**

DEAR DOCTOR—I have so often appealed to the profession in the South upon the subject of the importance of developing the medical literature of our section, that it is not without fear of being tiresome that I again address my medical brethren.

For seven years I have labored to establish a practical and useful medical journal, as a medium through which the busy practitioner could communicate valuable facts, and in which he could post himself in the progress of the profession. In this enterprise I have succeeded, though not without many and trying difficulties and heavy pecuniary sacrifices.

The obstacles to success in Southern journalism are so great that failure has heretofore been the uniform result of these enterprises.

Amongst the leading difficulties encountered is the want of pride and interest by the profession for their own section, and consequent neglect of their own journals. To this may be added a carelessness in renewing their subscriptions, so that it requires of the journalist the same expense, labor, and persuasion to hold a list that it did to procure it in the outset. Many never renew—others never pay; and, as the expenses of publication are cash and invariable, the gauntlet of failure must be run each succeeding year. Good publishing houses in the South are rarely to be found. Printing is expensive, and promptness in the execution of the work the exception to the rule. Ignorance of printers as to medical symbols and technicalties makes the work undesirable to them, and gives the editor immense trouble in the matter of proof. These, with other difficulties unknown to our readers, tax the patience and endurance of the editor, and subjects him often to undeserved criticism and complaint.

For seven years we have borne up under these trials, amidst the harassments of a large practice, no small portion of the income from which has been devoted to the journal enterprise. In all this our leading incentives had been the love of the profession, and the desire to develop the medical literature of our section.

In January of last year we decided, at the suggestion of friends, to reduce the size and price of the journal, as it was believed that it would suit a majority of practitioners; that the list would be increased, and that the expenses of publication being reduced, the journal would do better; and, by giving more attention to condensation of matter, the readers would lose nothing by the arrangement. In accordance with this idea, the RECORD was changed to its present form.

Unable, on account of pressing professional engagements, to look after the details properly, we procured the consent of Dr. R. C. Word, an experienced practitioner, a ready writer, and safe business man, to assist us and to take charge of the business management of the office. Under this arrangement the journal has been steadily gaining ground. Plain, cheap, brief and practical, it is everywhere welcomed by the busy practitioner, and for the first time has proven more than self-sustaining. Under these encouragements, we have added four additional pages of reading matter to each number, with other improvements. The journal may now be regarded as entering upon a new and permanent career of success and usefulness.

Yet we here wish to say to our medical brethren, what their good sense will readily confirm, that this assurance of success is, after all, based upon the hope of the co-operation of our subscribers and the busy practitioners throughout the land. Send us brief communications, practical notes, suggestions and formulæ; *and don't neglect an opportunity of sending us a new subscriber whenever you can.* Bring the matter before your medical acquaintances, and before your medical society, if you have one.

The editors are doing all they can. Circulars and appeals are sent out, but do little good, as they are soon forgotten, or are thrown aside without notice. The editors have no doubt that if they could see and talk with medical men personally, they could prevail with a majority to take the journal; but this they cannot do. Therefore we ask our readers to assist in extending our circulation, that the RECORD may be made more and more interesting and useful, that the busy practitioner may be benefitted, the progress of medical science advanced, and the medical literature of our section be developed.

Yours,

T. S. POWELL, M.D., Senior Editor.

### *EDITORIAL AND MISCELLANEOUS.*

☞ All communications relating to the business of THE RECORD, for the years 1877 and 1878, must be addressed to DR. R. C. WORD, Managing Editor Southern Medical Record, Atlanta, Ga.

☞ Brief and practical communications are solicited on all subjects pertaining to medicine; also reports of cases in practice.

☞ Send money by check, postal order or registered letter.

☞ Write your name, post-office, county and State plainly.

☞ The *Medico-Chirurgical Association,* of this city, unsolicited by the editors, has by resolution adopted our journal as the organ of the association, in which the reports and discussions of that body are to be published.

We appreciate the honor implied by this action of the society, and will, from time to time, report a brief of any matter likely to benefit or interest our subscribers; and if any of our readers take issue with the views expressed upon any important or practical point, they are at liberty to communicate the same through our pages, as it is by the free expression and interchange of views that information is imparted and progress made.

RECEIPTED.—Drs Lockwood Allison, S M Hogan, A B Loving, '77; N G West, R H Edwards, E Wheeler, J W Baker, W L Johnson, John Fale, J W Ethridge, J A Field, G W Allen, W H Lindsey, R J McMullen, T B Calling, N W McRee, E H Hurst, Tho J Hendley, R D Jacobs, G W Smith, '77; Tho B Meacham, J T Cleveland, A L East, Arnold & Quinn, Jno Hardamon, H T Shiell, 6 ms, W H Stewart, H J Walker, T W Spruell, J T & J F Alford, W T Gresham, M J Anderson, Gillespie & Payne, A Williamson, W L Posey, D Hagood, E R Young, B M Walker, '76 and '77; Henderson & Robertson, D G Hunt, E P Booth.

